# Intensive rice cropping drives shifts in abundance, activity, and assembly of root-associated methanotrophic community

**DOI:** 10.1093/femsec/fiaf112

**Published:** 2025-11-13

**Authors:** Sofía Croci-Bentura, José A Terra, Lucia Ferrando

**Affiliations:** Laboratorio de Ecología Microbiana Medioambiental, Departamento de Biociencias, Facultad de Química, Universidad de la República, Av. Gral. Flores 2124, 11800, Montevideo, Uruguay; Programa de Posgrados de la Facultad de Química, Universidad de la República, Av. Gral. Flores 2124, 11800, Montevideo, Uruguay; Instituto Nacional de Investigación Agropecuaria, INIA-Uruguay, Ruta 8 km 281, Treinta y Tres 33000, Uruguay; Laboratorio de Ecología Microbiana Medioambiental, Departamento de Biociencias, Facultad de Química, Universidad de la República, Av. Gral. Flores 2124, 11800, Montevideo, Uruguay

**Keywords:** aerobic methane oxidation, crop rotations, intensification, *Methylocella*, *pmo*A gene, rice rhizosphere, *Stable Isotope Probing*

## Abstract

Rice is a staple crop relevant to present and future human feeding. However, these agroecosystems significantly contribute to greenhouse gas methane emissions. In Uruguay, a traditional low-intensity, reduced-tillage rice system alternates annual rice crops with pastures for livestock. We hypothesize that rice crop intensification impacts aerobic methanotrophic communities associated with rice roots, which are crucial in mitigating methane emissions. The *pmo*A gene abundance, methane oxidation potential (MOP), and methanotrophic community composition by 16S rRNA gene Illumina MiSeq (V4 region) allowed us to determine the dynamics of these communities in bulk and rhizospheric soils from continuous rice (CR) and rice-pastures (RP) rotations throughout the crop cycle. Results showed that rice crop intensification significantly affected MOP and *pmo*A abundance in both compartments. The tillering stage showed the greatest *pmo*A abundance and MOP. Rhizospheric methanotrophic communities from the CR and RP systems at flowering differed greatly. While *Methylocystis* dominated rhizospheric CR soil, *Methylocella* predominated in those from RP rotation. Active rhizospheric methanotrophic communities at flowering detected by ^13^CH_4_ DNA-SIP were dominated by distinct *Methylocystis*-affiliated ASVs in both cropping systems. However, other active genera were differentially enriched in the two contrasting cropping systems. These results suggest aerobic methanotrophs could be a microbial guild sensitive to crop intensification.

## Teaser

Rice crop intensification affects the composition, abundance, and activity of aerobic methanotrophic communities associated with rice roots.

## Introduction

Methane is a potent greenhouse gas with a key role in global climate change, contributing about 17% to global warming (IPCC 2014). A broad range of natural and anthropogenic sources emit methane. Agricultural activities are responsible for 50% of anthropogenic methane emissions; rice paddies account for about 20%, i.e. 31 Tg of CH_4_ per year, becoming one of the primary sources of methane (IPCC 2023). Methane emissions result from the balance between methane produced by anaerobic digestion of organic matter, with strict anaerobic methanogenic archaea responsible for methane production, and biological methane oxidation that could be held under aerobic or anaerobic conditions by different microorganisms, including bacterial and archaeal clades (Ettwig et al. 2010, Dedysh and Knief 2018, Evans et al. 2019, Guerrero-Cruz et al. 2021, van Spanning et al. 2022). High methane-emitting environments, such as rice paddies, are hotspots for aerobic methanotrophs (Lim et al. 2024). However, anaerobic methanotrophic microorganisms have also been detected in rice paddies, even in rice rhizosphere (Vaksmaa et al. 2016, 2017).

Rice methane emissions to the atmosphere occur not only through diffusion and ebullition but also are mediated by rice plants through specialized tissue, i.e. the aerenchyma, which facilitates gas exchange between the atmosphere and the roots (Malyan et al. 2016). The rice rhizosphere is an essential microbial hotspot in paddy soils for methane production and oxidation (Wei et al. 2019) due to root exudates and decaying root cells (Kuzyakov and Blagodatskaya 2015). Since almost 90% of methane is emitted through the rice plant (Conrad 2007), methanotrophs associated with rice plants are key players in regulating methane emissions.

Aerobic methanotrophs not only play an essential role in consuming the greenhouse gas methane but have also been suggested to serve as a key biotic factor in maintaining soil fertility in paddy soils due to their important role in soil organic carbon accumulation via the methanotroph-mediated synthesis of biomass (Sultana et al. 2022). The structure and activity of aerobic methanotrophic communities have been demonstrated to be affected by different but interrelated factors like temperature, pH, methane, oxygen, and carbon dioxide concentrations, as well as the N and C soil contents and agricultural practices production systems (Hanson and Hanson 1996, Malyan et al. 2016, Kharitonov et al. 2021, Lim et al. 2024). Biological methane oxidation in these agroecosystems is key in mitigating methane emissions. Aerobic methanotrophs are resilient to sporadic disturbances but vulnerable when these disturbances persist or increase in frequency (Lim et al. 2024). Their activity, thus the methane sink trait, could be significantly affected by changes in land use derived from anthropogenic activities. The incorporation of other crops, like soybean, maize, or pastures in rotation with rice has shown an impact on physicochemical and biochemical soil properties and rice yields (Macedo et al. 2021b, Benintende et al. 2008, Xuan et al. 2012) but also on the structure of bacterial (Xuan et al. 2012) or fungal (Maguire et al. 2020) communities from maize or mungbean-rice or rice-pastures rotation soils, respectively, when compared to the rice monoculture. Furthermore, some authors have found that root colonization by *Archaea* and *Bacteria* was strongly affected by crop rotation between flooded rice and upland maize (Breidenbach et al. 2017).

*Stable Isotope Probing* (SIP) is a powerful technique that links metabolic activity to taxonomic identity (Chen 2011). It has been extensively used to address different active microbial populations, particularly aerobic methanotrophs in various environments (Radajewski et al. 2000, Dumont et al. 2011, Paul et al. 2017, Zhang et al. 2020) and agricultural soils, including rice paddy fields (Mayumi et al. 2010, Hu and Lu 2015, Sultana et al. 2019). Although intensive agricultural production systems that include crop rotations have shown an effect on soil methanotrophic communities (Breidenbach et al. 2016, Jiang et al. 2022), the impact on total and active rhizospheric methanotrophic communities has been scarcely addressed.

Rice in Uruguay has been traditionally cultivated in rotation with perennial pastures during the last 50 years, alternating 2 years of rice with 2–4 years of perennial mixed pastures for livestock grazing. This particular integrated agroecosystem has contributed to sustained yield increases, high eco-efficiency, the conservation of natural resources, and notable environmental indicators, contrasting with other more intensive rice systems around the world (Deambrosi 2003, Pittelkow et al. 2016, Tseng et al. 2020, Castillo et al. 2021, Terra et al. 2023). Seeking productivity improvements, the intensification of soil use (shorter pastures’ phases or incorporation of other crops) has advanced in the Uruguayan rice-producing sector over the last decade (Macedo et al. 2021b). Sustainable high-yield rice systems intensification transitioning towards more intensive cropping systems constitutes a unique scenario scarcely addressed worldwide. Hence, studying the impact of contrasting rice production alternatives on aerobic methanotrophic communities established in the soil and associated with rice roots is crucial for redesigning other sustainable rice rotations. According to previous work, although rice soil only emitted CH_4_ under flooded conditions, differences among continuous rice, rice-pastures, and rice-soybean rotations were observed mainly at the tillering stage, when the abundances of methane cycle populations responded to the intensification of rice crop (Fernández-Scavino et al. 2022). Moreover, these rice rotation alternatives also impacted the abundance and structure of diazotrophic communities associated with rice plants (endophytic and rhizospheric) (Ghiazza et al. 2023).

This work aimed to study the impact of contrasting rice rotation systems on aerobic methanotrophic communities inhabiting rice rhizospheric and bulk soils from a long-term field experiment. We focused on two contrasting rice cropping systems: continuous rice (the most intensive, although not commercially used in Uruguay) and rice-pasture rotation (a low-intensity system, traditionally and extensively used in Uruguay). The abundance of the *pmo*A marker gene, the methane oxidation potential (maximum methane oxidation rate), and the methanotrophic community structure based on 16S rRNA gene amplicon sequencing were assessed at different crop growth stages. In addition, active rhizospheric methanotrophs from the two rice rotations at the flowering stage were evaluated by performing a ^13^CH_4_ DNA-SIP microcosm experiment combined with 16S rRNA gene amplicon sequencing.

## Materials and methods

### Field experiment and sample collection

The bulk and rhizospheric soils were sampled in 2019 from a field-scale experiment installed in 2012 at Paso de la Laguna, Experimental Station of the National Agricultural Research Institute (INIA), in Treinta y Tres, Uruguay (33° 16′ 23′' S; 54° 10′ 24′' W; 22 MASL). The field experiment was already exhaustively described in Ghiazza et al. (2023). Briefly, the site has a mesothermic humid climate. Soil at the site is classified as a Typic Argialboll according to USDA Soil Taxonomy (Durán et al. 2006) or as a Mollic Planosol according to the World Reference Base for Soil Resources (IUSS Working Group WRB 2022), with a silty clay loam texture (18% sand, 52% silt, 30% clay) in the A horizon and a slope lower than 0.5%. Before the experiment’s setup, the soil’s physicochemical properties were as follows: TOC, 14.2 g kg^−1^, TN 1.4 g kg^−1^, P Bray 7.0 µg g^−1^ and pH 5.7. A rice-pastures rotation system (2 years of rice and 3 years of pastures) was used for the previous 34 years. The long-term experiment evaluates different rice rotation systems under no-till with contrasting soil use intensity determined by the proportion of rice, pastures, and other crops in the rotation (Macedo et al. 2021a). The experiment was arranged in a randomized complete block design with three replicated plots and all phases of the rotations (crop and pastures) simultaneously present (Patterson 1964), with an area plot of 20×60 m. Two contrasting rotation systems were selected for this work (see the scheme of the rotation systems selected in Supplementary  Fig. S1). Continuous rice rotation is not a valid commercial production alternative in Uruguay, but it represents the most intensive rice cropping system. For each rotation system a single rice phase (R) was studied: (a) continuous rice (CR: rice every summer); (b) the first rice after a pasture from a rice-pasture rotation [RP: 2 consecutive rice crops followed by 3.5 years of a mixed pasture of tall fescue (*Festuca arundinacea*), white clover (*Trifolium repens*), and birdsfoot trefoil (*Lotus corniculatus*)]. In the two rotations, ryegrass (*Lolium multiflorum*) and/or *Trifolium alexandrinum* L. were used as cover crops during fall-winter between cash crops (Macedo et al. 2021a). The Merin rice variety (*Oryza sativa, subtype Indica)* was no-till drill-seeded on October 25, 2019, emerged on November 15, and was flooded on December 2, approximately 4 weeks after emergence at the rice V4-V5 growth stage. Nitrogen (N) fertilizer was applied as urea in two growth stages, at V4 tillering (December 2nd, upland conditions) and at R0 panicle initiation (December 30th, flooded soil). Annual nitrogen rates were 63–86 kg ha^−1^ of N in rice in RP, while in CR it was 140 kg ha^−1^ of N, following national guidelines and recommendations (Macedo et al. 2021a).

The samplings were performed during the 2019–2020 growing season, eight years after the experiment was set up. Bulk soils and rice plants were collected from each of the three blocks (replicates) of each rotation system at different phenological stages of rice growth. Bulk soil was sampled at pre-seeding (PS; October 23rd, 2019; upland conditions), tillering (T; December 11th, 2019, 30 days after emergence, nine days after N fertilization and flooding), and flowering (F; February 12th, 2020; flooded) stages. At the same time, rice plants were sampled at tillering (flooded), panicle initiation (R1) (PI; January 8th, 2020, nine days after N fertilization; flooded), and flowering (flooded) stages. Ten or fifteen bulk soil cores (0–10 cm depth) were randomly taken from each plot using an auger for coring between lines, homogenized, and composited into a single sample from each replicate. For each plot, 6 to 10 rice plants with their respective soil blocks surrounding the roots (approximately 20×20×10 cm) were randomly collected, avoiding the border effect, and then refrigerated and transported to the lab for further processing.

### Sample processing

Bulk soil samples (S) were sieved through a 6 mm mesh to remove stones and plant debris, then temperature-controlled dried at 30°C, and subsequently sieved through a 3 mm mesh. Fresh and dry bulk soils were stored at 4°C and room temperature for methanotrophic potential activity and physicochemical analyses, respectively, and fresh samples were frozen at −70°C for further molecular analysis.

Rice plants sampled from rice crops in the CR and RP cropping systems (three blocks) were processed. Rhizospheric soil samples (Rh) were manually collected using a sterilized spatula from the central, densely rooted zone of each rice plant. Rh samples were stored at 4–8°C for no more than 48 h for methane oxidation potential (MOP) and physicochemical analyses, and at −70°C for DNA extraction and molecular studies.

### Physicochemical characterization of bulk and rhizospheric soils

The humidity of rhizospheric and bulk soils as well as the pH of suspensions of bulk and rhizospheric soils in distilled water, were determined according to Tan (2005). The N-NH_4_^+^ content and active soil carbon were measured for bulk and rhizospheric soils from the different growth stages. The N-NH_4_^+^ content was determined from fresh soil 2 mol L^−1^ KCl extractions by a spectrophotometric technique according to Rhine et al. (1998). Active soil carbon or soluble carbon, hereafter POXC (permanganate-oxidizable carbon), was determined for air-dried bulk and rhizospheric soils by oxidation of soils with 0.2 mol L^−1^ potassium permanganate solution in 1 mol L^−1^ CaCl_2_ according to Weil et al. (2003). Two replicates were performed for both determinations.

### DNA extraction and abundance of the *pmo*A gene by qPCR

According to the manufacturer’s instructions, 0.25 g of bulk or rhizospheric soil samples were extracted using the DNeasy PowerSoil Pro Kit (QIAGEN®, Hilden, Germany). Then, DNA was quantified with the Qubit™ dsDNA HS Assay Kit using a Qubit® 2.0 Fluorometer (Invitrogen). The abundance of the *pmo*A gene was determined by real-time PCR (qPCR) using a Rotor-Gene® 6000, model 5-Plex (CORBETT Research, Sidney) as described in Fernández-Scavino et al. (2022). The reaction mixture contained 1 µL of diluted (one 10-fold) template DNA, 1 µmol L^−1^ of primer A189gcf (5′-GG(AGCT) GAC TGG GAC TTC TGG-3′) and mb661r (5′-CCG G(AC)G CAA CGT C(CT)T TAC C-3′) (Costello and Lidstrom 1999), and 5 µL of Rotor-Gene SYBR Green PCR Mastermix (QIAGEN®, Hilden, Germany). The thermal cycle was as follows: an initial step at 95°C for 5 min followed by 40 cycles of 95°C for 5 s, 60°C for 10 s, and 82°C for 1 s for fluorescence acquisition. A melting curve was performed from 65 to 94°C. All samples were amplified in duplicate, and the standard curves were generated for each qPCR run in triplicate for each standard point (2.67 to 2.67 × 10^6^  *pmo*A copies µL^−1^) prepared from the strain *Methylogaea oryzae* E10^T^, DSM 23452. Triplicates of no-template controls were included in each run as a negative control. The results were expressed as *pmo*A copy number per g of dry soil weight.

### MOP in bulk and rhizospheric soil slurries

The MOP was measured (using two slurry incubation replicates per biological replicate plot) for bulk and rhizospheric soils at different crop stages (Ferrando and Tarlera 2009, Fernández-Scavino et al. 2022). Firstly, fresh soil (equivalent to 5 g of dry weight) was preincubated with 20 mL of sterilized distilled water in 120 mL glass vials with cotton plugs at 28°C in the dark under oxic conditions to allow the depletion of endogenous organic substrates that could be present in the soil samples and consume the oxygen interfering with MOP determination. After the oxic incubation, the resulting slurries’ vials were closed with butyl stoppers under an air atmosphere containing 7% CH_4_ (v/v) and incubated in darkness at 28°C and 150 rpm in an orbital shaker. Methane consumption was monitored by sampling the headspace and subsequent GC-TCD analysis (GC-2014, Gas Chromatograph, Shimadzu; column temperature 35°C, detector temperature 100°C, and a Molecular Sieve 13× column SRI, Torrance, CA, USA). A standard curve was performed comprising five different methane concentrations (three technical replicates each), ranging from 356 to 3206 nmol mL^−1^ that were prepared by diluting pure CH_4_ (99.995%; AGA) in vials closed with butyl stoppers to achieve the desired concentrations. Methane concentration was calculated from the standard curve by interpolating the methane areas retrieved. Finally, potential CH_4_ oxidation rates were calculated using linear regression analysis of methane consumption over time.

### ^13^CH_4_ Stable Isotope Probing (*DNA-SIP*) experiment and microcosms incubation processing

A time course experiment was set up for rice rhizospheric soils from CR and RP rotations at the flowering stage. For rhizospheric soils from each cropping system (CR and RP) at the flowering stage, a pool of the three replicated plots was used for the DNA-SIP experiment. Microcosm incubations were prepared similarly to what was described for the MOP (5 g of soil dry weight; oxic preincubation 72 h at 28°C at 150 rpm; addition of 7% CH_4_ (v/v); incubation at 28°C at 150 rpm). Four replicates were set up and added with ^13^CH_4_ (99% ^13^C; Sigma–Aldrich, cat. no. 490229–1 L), and another four with ^12^C-CH_4_ (control incubations; CH_4_ 99.995%; AGA) for each rice rotation. Methane consumption was monitored daily by sampling the headspace and subsequent GC-FID analysis (GC-2014, Gas Chromatograph, Shimadzu; column temperature 55°C, detector temperature 140°C, and a Porapak Q column). A total of three pulses of labeled (or control) methane were added to the vials until sufficient ^13^C-CH_4_ was incorporated into the biomass, as described by Chen (2011). A destructive sampling of two replicates for each rice rotation system was performed at 9 and 14 days of incubation; however, only the 9-day time point vials were further processed, as this time point was sufficient for labeling DNA. Rhizospheric soil slurries were centrifuged, and the pellets were kept at −70°C until DNA extraction and quantification were performed under the same conditions explained before.

### Ultracentrifugation in CsCl gradient and gradient fractionation

The ultracentrifugation protocol was based on that described by Neufeld et al. (2007). The extracted DNA of rhizospheric soils from the two replicates (2 μg) was mixed with a gradient buffer (100 mmol L^−1^ Tris-HCl; 100 mol L^−1^ KCl; 1.0 mmol L^−1^ EDTA, pH 8.0) and a CsCl (Sigma–Aldrich, C4036; purity ≥98%) solution 7.163 mol L^−1^ to obtain a final solution with a density of 1.725 g mL^−1^. Eight-mL polyallomer tubes were carefully filled and sealed according to the manufacturer’s recommendations. Ultracentrifugation was performed using in a Sorvall MX 150+ ultracentrifuge, using a fixed-angle rotor (S80-AT3-2014; angle 30°), at 177 000 *g* for 40 h at 20°C under vacuum, with maximum acceleration without break. After ultracentrifugation, gradient fractionation was performed by pumping sterilized distilled water colored with violet crystal dye using a low-flow peristaltic pump (Gilson Miniplus 3) at a flow rate of 680 μL min^−1^. Fifteen fractions (∼450 μL each) were retrieved from each fractionation. Gradient formation was confirmed by measuring the refractive index corrected by temperature (nD-TC) in each fraction using an ABBE Boeco digital refractometer and calculating their buoyant densities from a standard curve prepared previously using a CsCl solution (range 1.850–1.650 g mL^−1^).

### DNA extraction and methanotrophic ^13^C-DNA enrichment in ^13^C-CH_4_ incubations

DNA was retrieved from each fraction according to Neufeld et al. (2007) using PEG 6000 solution and glycogen (Invitrogen® 5 mg mL^−1^) precipitation, followed by purification with 70% ethanol. DNA was dissolved in 30 μL of sterilized bidistilled water and quantified by Qubit® as described previously. Methanotrophic enrichment in heavy fractions of ^13^C-CH_4_ incubations was verified by measuring DNA concentration in each fraction and quantifying the marker gene *pmo*A by qPCR, as previously described. Also, fractions from control incubations (^12^C-CH_4_) were quantified. The relative abundance of *pmo*A copies in each fraction divided by the total number of *pmo*A copies in each tube was calculated to evaluate the distribution of the marker gene throughout all the fractions.

### 16S rRNA gene sequencing by Illumina MiSeq and data processing

Microbial communities enriched in light (L) and heavy (H) fractions from rhizospheric soil of control (^12^C-CH_4_) and labeled (^13^C-CH_4_) slurry incubations, from continuous rice (CR) and rice-pastures (RP) rotations, were analyzed by *Bacteria* 16S rRNA gene amplicon sequencing (Illumina MiSeq, 250 bp paired-end, V4 region). DNA from CR and RP’s original rhizospheric soils was also sequenced. The fractions to be sequenced were selected based on the *pmo*A relative abundance, DNA concentration, and the densities of the fractions (Deng et al. 2019). Before sequencing, the labeled DNA from H-^13^C-CH_4_ fractions was concentrated using a Microcon® 100 DNA fast flow centrifugal filter (Merck®).

The V4 variable region of the 16S rRNA gene was amplified using primers 515F and 806R (Caporaso et al. 2011) according to standard procedures in the MrDNA lab facilities (www.mrdnalab.com, Shallowater, TX, USA). An Illumina DNA library was prepared from the pooled and purified PCR product (using calibrated Ampure XP beads). Sequencing was performed at MR DNA on an Illumina MiSeq sequencer following the manufacturer’s guidelines. Raw data were deposited in the NCBI Sequence Read Archive (SRA) under BioProject number PRJNA1210543.

The retrieved Illumina MiSeq raw data were processed in the R environment using DADA2 (Callahan et al. 2016). The forward and reverse reads were checked, filtered, and trimmed (200 bp). The package truncQ (quality threshold = 2) was used and reads with ambiguous bases or a maximum expected error (maxEE) greater than 2 were removed. The DADA2 pipeline included dereplication, removal of erroneous reads, merging of R1 and R2 reads, and depuration of contigs. Chimeric ASVs were removed using the removeBimeraDenovo function from dada2 package. Afterward, an ASV table containing the abundance of each ASV was constructed, and the SILVA reference database (version SILVA 138.1; McLaren 2020) was used for taxonomic assignment. ASVs corresponding to mitochondria, chloroplast, *Eukarya*, or *Archaea* domains and those not assigned to any taxa were removed using the phyloseq package (McMurdie and Holmes 2013). Lastly, taxa represented by fewer than 3 reads in the whole dataset were also removed. Data normalization was performed using the rarefy_even_depth function, and ASV relative abundances were calculated. Alpha diversity was analyzed by retrieving the richness, Shannon–Weaver, Chao1, and Pielou’s equitability indices (packages phyloseq version 1.40.0 and ggplot2 (version 3.3.6, Whickham 2016) in RStudio. Beta diversity was addressed by PCoA ordination analysis using the Bray–Curtis distance. Putative known methanotrophic sequences were selected from the total ASV table based on those reported by other authors (Dedysh and Knief 2018, Guerrero-Cruz et al. 2021, van Spanning et al. 2022) to compare the total methanotrophic communities from rhizospheric soils with those active in the SIP experiment. Using the package ampvis2 (2.7.32, Andersen et al. 2018), a heatmap was obtained for the relative abundance of ASVs (square-root-transformed). Differential Abundance Analyses were performed on the methanotrophic genera enriched in heavy (H) fractions from ^13^CH_4_ incubation in the SIP Experiment. The log2 fold-change analysis was performed with DESeq2, according to Love et al. (2014).

### Statistical analyses

All statistical analyses were performed using R software with RStudio version 4.2.1. Physicochemical parameters, MOP values, abundances of the *pmo*A gene, and diversity indices comparisons were performed using ANOVA, Tukey’s Test, and the non-parametric Kruskal–Wallis test (for diversity indices comparison and some physicochemical parameters’ comparisons). When corresponding, Dunn’s Test was used for multiple comparisons (α = 0.05) or the Mann–Whitney *U* Test for pairwise comparisons (α adjusted by the Bonferroni method). Correlation among different parameters (physicochemical, *pmo*A abundance, and MOP) was evaluated by a Principal Component Analysis (PCA), and Spearman correlation coefficients were calculated using the *stats* and *ggbiplot* packages. An ANOSIM analysis was used to compare the microbial and methanotrophic community structures retrieved by 16S rRNA gene amplicon sequencing, since variance homogeneity was not confirmed.

## Results

### Characterization of bulk and rhizospheric soils

Permanganate-oxidizable carbon (C-POX) did not show significant differences for crop rotation, crop stage, or compartment (Supplementary  Table S1), according to the non-parametric Kruskal-Wallis statistical analyses (*P*-values 0.3113, 0.09568, and 0.8618, respectively). The N-NH_4_^+^ content was not significantly different between crop rotations and between compartments but showed significant differences among crop stages (Supplementary  Table S1; Kruskal–Wallis, *P*-value= 9.705×10^−7^). According to the *post hoc* pairwise comparisons performed through the Mann-Whitney U test (α= 0.00625, adjusted with the Bonferroni method), N-NH_4_^+^ content at tillering was significantly higher than at flowering for both rhizospheric and bulk soils (*P*-values 3.1×10^−5^ and 3×10^−4^, respectively). Additionally, no relevant statistical differences were observed for pH values (Supplementary  Table S1).

### Bulk and rhizospheric soils from the CR system had greater *pmo*A gene abundances

**Figure 1.**
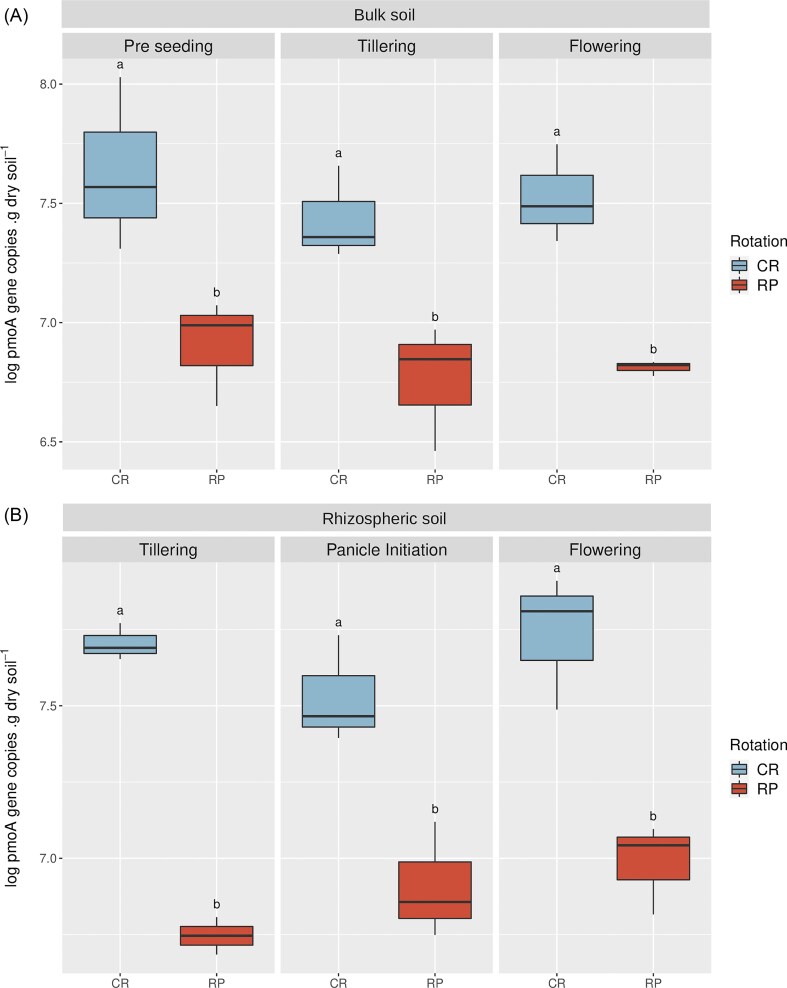
Dynamics of *pmo*A gene abundance in bulk (A) and rhizospheric (B) soils throughout the crop season. For each compartment, different letters indicate significant statistical differences (*n* = 3) in log_10_  *pmo*A copies g of soil dw^−1^ according to ANOVA and Tukey’s Test performed for bulk (*P*-value= 3.62×10^−5^) or rhizospheric soils (*P*-value= 1.95×10^−7^). CR: Continuous Rice. RP: Rice- Pastures rotation.

The mean bulk soil *pmo*A abundances were between 6.42 × 10^6^ and 5.48 × 10^7^ copies g^−1^ dw. Similarly, the rhizospheric soils had *pmo*A abundances ranging from 5.61 × 10^6^ to 5.88 × 10^7^ copies g^−1^ dw. The ANOVA indicated that the *pmo*A gene abundances between both compartments throughout the crop cycle did not show significant differences (*P*-value = 0.189). Conversely, the CR system had significantly higher abundances of this marker gene than RP rotation regardless of the crop stage sampled, not only for bulk soils (CR means between 2.92 × 10^7^ and 5.48 × 10^7^ copies g^−1^ dw; RP means between 6.42 × 10^6^ and 8.68 × 10^6^ copies g^−1^ dw; ANOVA and Tukey Test, *P*-value = 3.62×10^−5^) but also for rhizospheric soils (CR means between 3.60 × 10^7^ and 5.88 × 10^7^ copies g^−1^ dw; RP means between 5.61 × 10^6^ and 1.00 × 10^7^ copies g^−1^ dw; ANOVA and Tukey Test, *P*-value = 1.95×10^−7^), and no significant interaction between crop stage and crop rotation was found in each compartment (Fig. 1; *P*-values 0.211 and 0.977 for rhizospheric and bulk soils, respectively).

### The tillering stage showed the most significant MOP, and CR differentiated from RP rotation

**Figure 2.**
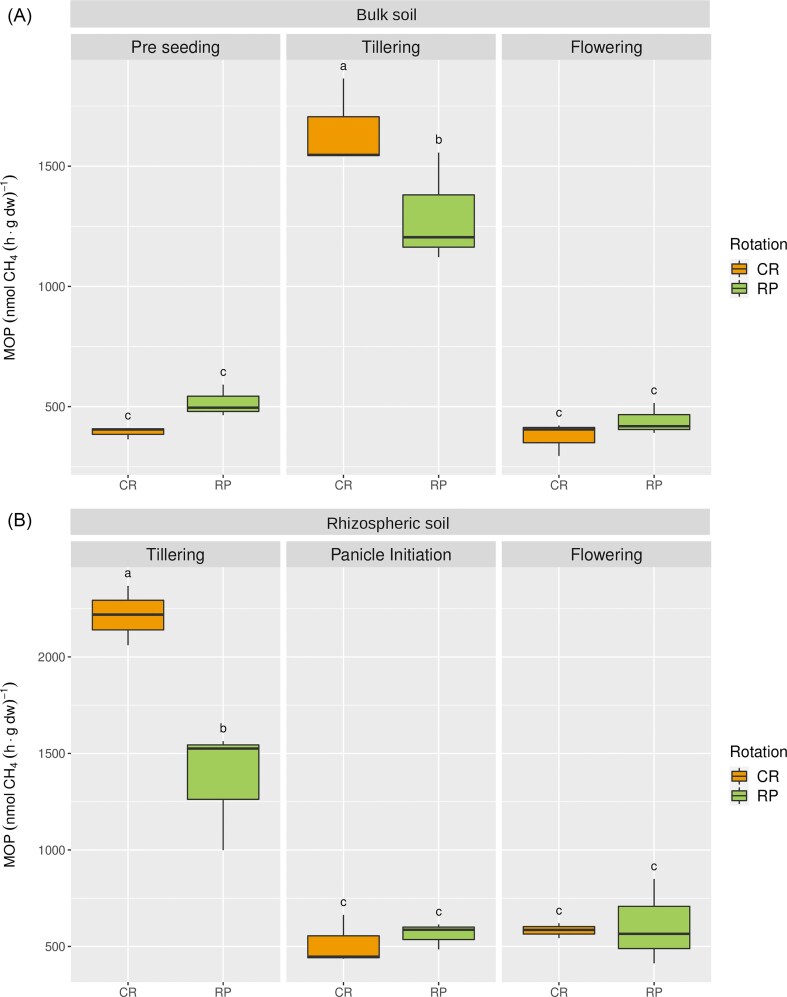
Dynamics of MOP in bulk (A) and rhizospheric (B) soils throughout the crop season. According to ANOVA and Tukey’s test performed, a significant crop stage * crop rotation interaction was found for bulk (*P*-value= 0.00147) and rhizospheric soils (*P*-value= 0.00129). Different letters indicate statistically significant differences (*n* = 3). CR: Continuous Rice. RP: Rice-Pastures rotation.

Methane oxidation rates were determined for slurries incubations from bulk soils at pre-seeding, tillering, and flowering and rhizospheric soils at tillering (T), panicle initiation (PI), and flowering (F) stages (Fig. 2). Bulk soils showed MOP mean rates between 373 and 1651 nmol CH_4_ (h g dw)^−1^. MOP mean rates for rhizospheric soils were between 583 and 2216 nmol CH_4_ (h g dw)^−1^, corresponding to PI and T stages. The statistical comparisons of the MOP revealed that a significant crop stage * crop rotation interaction was found for bulk soil rates (ANOVA Tukey test, *P*-value= 0.00147; Fig. 2A) and rhizospheric soil rates (ANOVA and Tukey test, *P*-value= 0.00129; Fig. 2B). The bulk and rhizospheric soils had the greatest MOP rates at the tillering stage. For both compartments, MOP rates from CR were higher than those from RP rotation at this stage (Fig. 2). Additionally, rhizospheric soils had a significantly greater MOP than bulk soils (ANOVA and Tukey Test, *P*-value= 0.000622). Additionally, the Spearman correlation analysis performed (Supplementary  Fig. S2) showed a strong positive correlation between MOP and N-NH_4_^+^ content (r = 0.73, *P* = 1.4 × 10^−5^) and a moderate negative correlation between MOP and pH (r= −0.39, *P* = 0.02).

### 16S rRNA gene amplicon sequencing by Illumina MiSeq

DNA extracted from rice rhizospheric soils from CR and RP rotation was sequenced. For the 20 samples sequenced (rhizospheric soils and heavy and light fractions from ^12^CH_4_ and ^13^CH_4_ incubations), 1 209 408 high-quality reads with a mean length of 252 bp in the V4 region of the 16S rRNA gene were retrieved. After analysis (the remaining number of reads after processing steps in Supplementary  Table S2), 3331 ASVs were obtained, with 3282 belonging to the *Bacteria* domain. Those ASVs not affiliated with *Bacteria* or belonging to mitochondria and chloroplast were not further considered. After normalization, 37 202 reads and 3133 ASVs per sample were obtained. A total of 2693 ASV were found in the different SIP fractions sequenced. The rarefaction curves showed a plateau in richness (Supplementary  Fig. S3), indicating that good coverage of bacterial diversity was retrieved.

### Evaluation of the ^13^CH_4_  *DNA-SIP* experiment

A SIP experiment was conducted through microcosm slurry incubations of rhizospheric soils from rice plants of CR and RP rotation systems at the flowering stage under oxic methanotrophic conditions described for MOP determinations. The methane incorporation reached between 74 and 89 µmol per gram of soil for the control (^12^C-CH_4_) and ^13^C-labeled methane (^13^C-CH_4_) microcosm incubations, adequate according to Neufeld et al. (2007). The use of a 30°C fixed-angle rotor resulted in a successful separation of labeled and unlabeled DNA, which was detected in fractions with buoyant densities between 1.69 and 1.72 g mL^−1^ for light fractions (^12^C) and between 1.72 and 1.80 g mL^−1^ for heavy fractions (^13^C).

**Figure 3.**
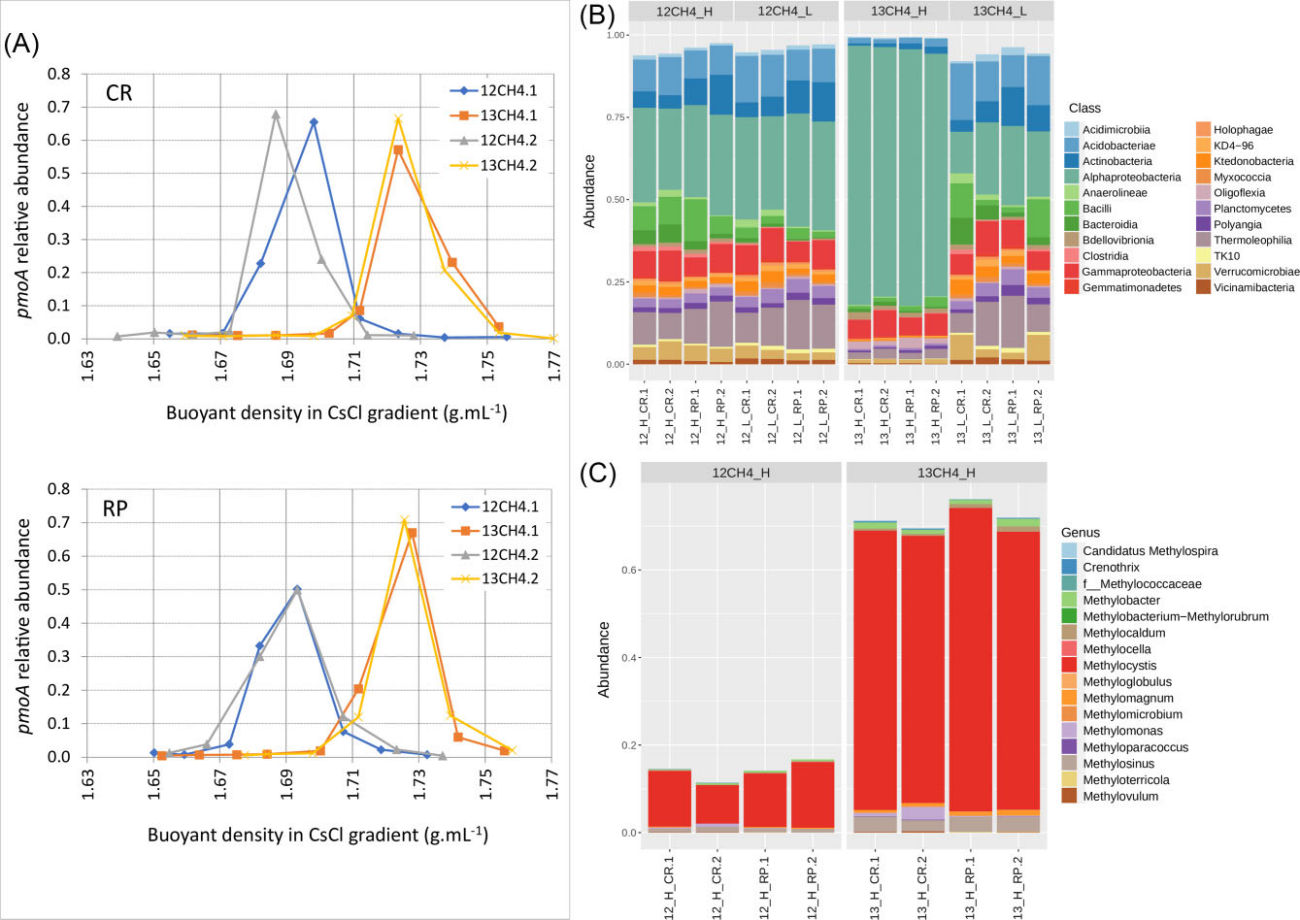
^13^CH_4_ DNA-SIP experiment for rhizospheric soils from continuous rice (CR) and rice-pastures (RP) rotations at the flowering stage. (A) Methanotrophic enrichment, as *pmo*A relative abundance in each fraction, obtained across the different buoyant densities in CsCl gradient fractionation. (B) Bacterial community composition at the Class level (16S rRNA gene amplicon-sequencing Illumina MiSeq; relative abundances >5%) for heavy (H) and light (L) fractions obtained from ^13^CH_4_ and control (^12^CH_4_) slurry incubations. (C) Abundance of methanotrophic genera detected in heavy (H) fractions from ^13^CH_4_ and control (^12^CH_4_) slurry incubations.

The relative abundance of the *pmo*A gene was determined in the different fractions, as is shown in Fig. 3A. A sharp methanotrophic enrichment was observed in heavy fractions for ^13^C-CH_4_ incubations (fractions 7, 8, and/or 9) and in light fractions in ^12^C-CH_4_ control incubations (fractions 10 and 11). 16S rRNA gene amplicon sequencing of selected fractions (or pool of fractions) confirmed that bacterial communities from fraction H-^13^C-CH_4_ distinguished from those from L-^13^C-CH_4_, L-^12^C-CH_4_, and H-^12^C-CH_4_, showing a significant enrichment of the class *Alphaproteobacteria* (Fig. 3B). PCoA analysis (Supplementary  Fig. S4) combined with ANOSIM analysis revealed that total bacterial communities from 13-H-CH_4_ fractions were significantly different than those from the remaining fractions (ANOSIM, *P*<0.1). The bacterial community structure of non-incubated rhizospheric soils was significantly different from that of 12-L fractions (ANOSIM *P* = 0.025), regardless of whether labeled or unlabeled methane was used (Supplementary  Fig. S4). However, there were no significant differences among the bacterial communities from 13-L fractions, 12-L fractions, and those from rhizospheric soils. A similar result was obtained when comparing 12-L and 12-H fractions. Additionally, the Richness, Equitability, Shannon, and Chao1 indices calculated were significantly lower for 13-H-CR and 13-H-RP compared to the remaining fractions (13-L, 12-L, and 12-H) and the original rhizospheric soils (ANOVA and Tukey test, *P*-value<0.01; Supplementary  Table S3) denoting the dominance of the group of bacteria enriched.

When methanotrophic communities were analyzed, not only the composition but also the relative abundance of methanotrophic ASVs in the total bacterial community showed significant enrichment of these bacteria in heavy fractions from ^13^C-CH_4_ incubations, i.e. 13-H-CR and 13-H-RP (Fig. 3C). The methanotrophic relative abundance in heavy fractions from ^13^C-CH_4_ incubations was higher than 70% for all samples, whereas it represented about 11%–17% of total bacterial ASV in L and H fractions from ^12^C-CH_4_.

### Active bacterial and methanotrophic communities detected by ^13^CH_4_*- DNA-SIP* experiment of rhizospheric soil from RP and CR rice cropping systems at flowering stage

**Figure 4.**
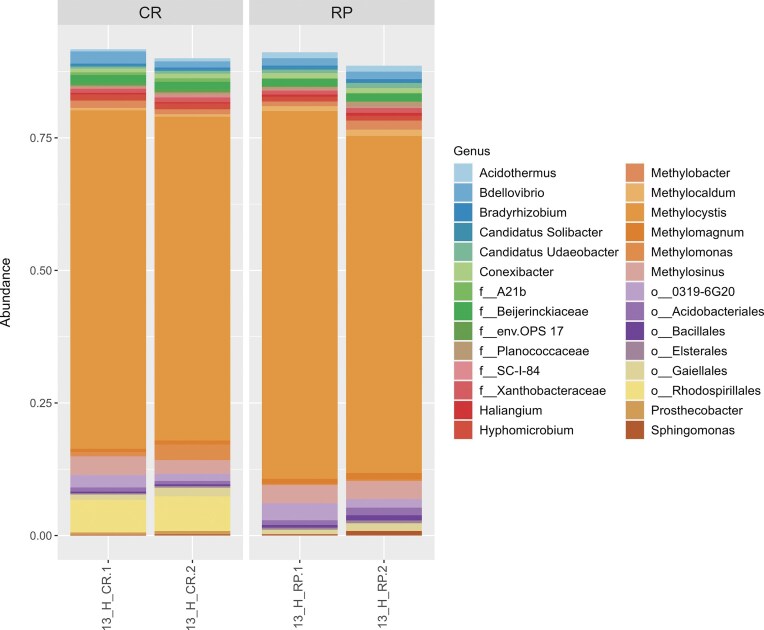
Active rhizospheric bacterial communities from continuous rice (CR) and rice-pastures (RP) rhizospheric soils at the flowering stage detected in heavy (H) fractions by ^13^CH_4_ DNA-SIP and 16S rRNA gene amplicon-sequencing. Dominant active genera (relative abundance >5%) are shown.

DNA-SIP and 16S rRNA gene amplicon sequencing from rhizospheric soils of CR and RP cropping systems revealed that the active bacterial communities under microcosm aerobic methanotrophic conditions were similar. Alpha diversity analyses did not show significant differences between CR and RP active bacterial communities (Supplementary  Table S3). Venn diagrams (data not shown) indicated that 150 ASVs were shared between CR and RP, whereas 52 and 71 ASVs were exclusive to CR and RP, respectively. Twenty-eight main genera, or taxa classified at the genus level, were dominant members of the active total bacterial communities, exhibiting relative abundances >5% (Fig. 4). Methanotrophic genera represented more than 70% of the total active bacterial communities, comprising type II alphaproteobacterial genera *Methylocystis* (the dominant genus in CR and RP cropping systems), *Methylosinus*, and type I gammaproteobacterial genera *Methylocaldum, Methylobacter, Methylomagnum*, and *Methylomonas*. Other genera or taxa non-methanotrophic like *Hyphomicrobium and Bradyrhizobium (class Alphaproteobacteria), Bdellovibrio* (phylum *Bdellovibrionota*), unclassified members of the families *Beijerinckiaceae, Xanthobacteraceae* (order *Hyphomicrobiales*), and the order *Rhodospirillales* were also found active. Additionally, members of the phyla *Myxococcota* (*Haliangium*), *Actinomycetota* (*Acidothermus, Conexibacter*), *Acidobacteriota* (*Ca*. Solibacter; unclassified Acidobacteriales), *Bacillota* (unclassified *Planococcaceae*; unclassified *Bacillales*), and *Verrucomicrobiota* (*Ca*. Uaeobacter) were found among the more abundant active bacteria in the microcosm under aerobic methanotrophic conditions.

**Figure 5.**
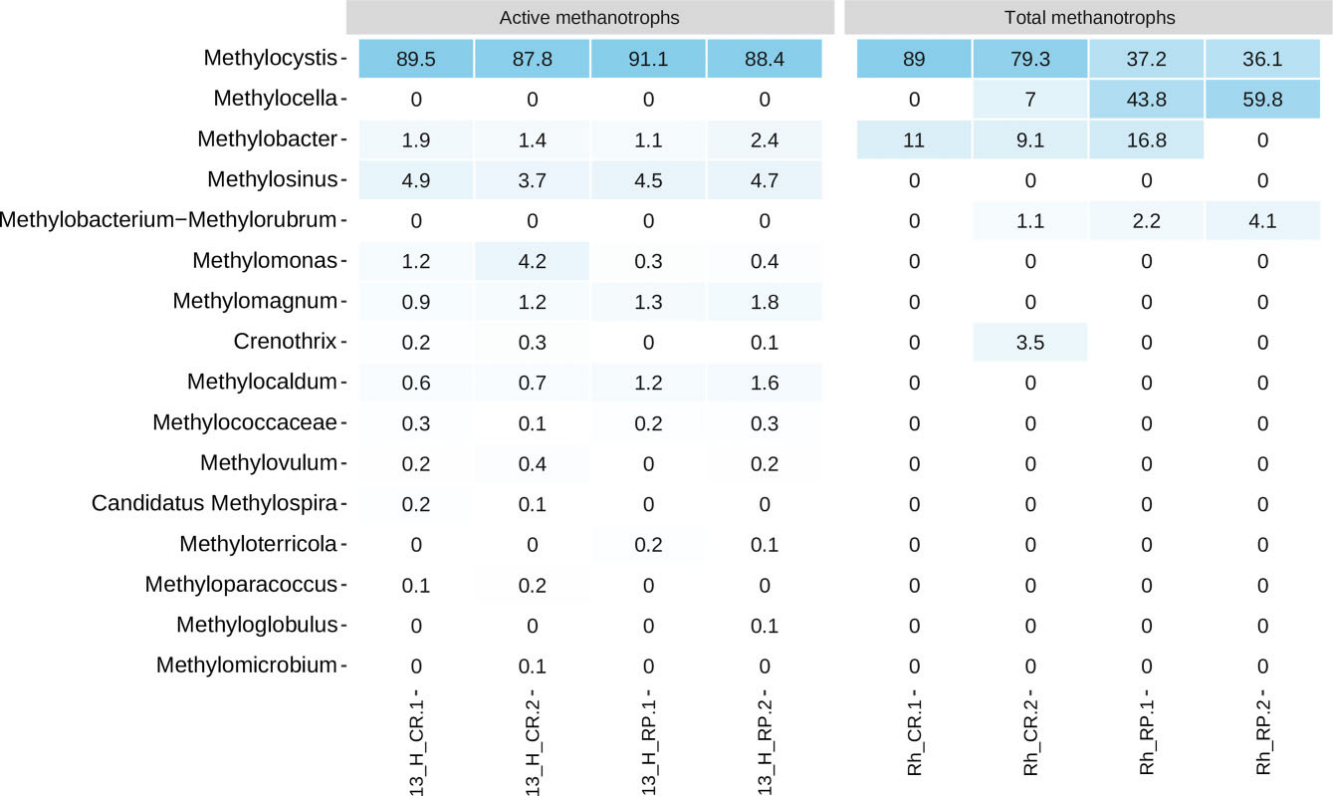
Total and active methanotrophic communities from continuous rice (CR) and rice-pastures (RP) rhizospheric soils at the flowering stage. Relative abundance heatmap of dominant methanotrophic genera (relative abundances >5%; mean values *n*=2) inhabiting original rhizospheric soils (Rh) and detected by ^13^CH_4_ DNA-SIP in heavy fractions (H).

After subsetting the ASVs corresponding to known methanotrophs, the relative abundances of active methanotrophic populations encountered in the 13-H-CH_4_ fractions showed that *Methylocystis* was greatly enriched under the microcosm conditions assayed, ranging from 87.8% to 89.5% of the relative abundance. Additionally, *Methylosinus* (3.68%–4.9%), also a type II methanotroph, was the next most abundant member among the active methanotrophic communities. The type I methanotrophs genera *Methylomonas* (0.3%–4.2%), *Methylobacter* (1.1%–2.4%), *Methylomagnum* (0.9%–1.8%), and *Methylocaldum* (0.6%–1.6%) were also detected among the dominant members of active methanotrophic communities at the flowering stage. Other genera enriched in lower relative abundances are shown in Fig. 4 and Fig. 5.

**Figure 6.**
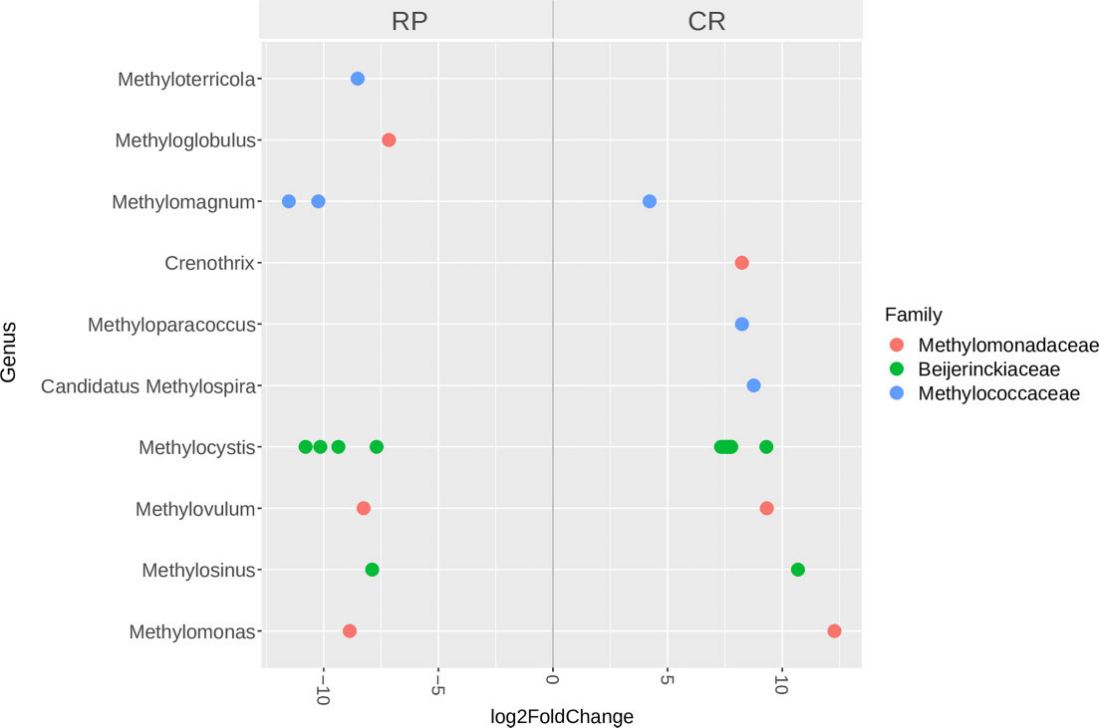
Differential abundance analysis for active rhizospheric methanotrophic genera detected in heavy fractions of ^13^CH_4_ slurry incubations for CR and rice-pastures (RP) at the flowering stage. Methanotrophic genera showing significant differential abundances (α=0.05) are shown at an ASV level. The log2 fold change shown was calculated by comparing CR with RP.

A differential abundance analysis compared the active methanotrophic communities from CR vs. RP cropping systems (Fig. 6). The results showed that *Crenothrix, Methyloparacoccus*, and *Ca*. Methylospira were significantly more abundant in active rhizospheric methanotrophic communities from the CR rotation, whereas *Methyloterricola* and *Methyloglobulus* were significantly enriched in those from the RP cropping system. Additionally, a particular behavior was observed for ASVs affiliated with *Methylocystis*. Although this genus was the most enriched in 13-H-CH_4_ fractions from both cropping systems, some ASVs were more abundant in active methanotrophic communities from CR rhizospheric soils, while others were more abundant in those from RP. Similar findings were observed for the genera *Methylomagnum, Methylovulum, Methylosinus*, and *Methylomonas* (Fig. 6).

### Rhizospheric methanotrophic communities from CR and RP rotation at flowering were different

Methanotrophic genera accounted for 1.1% and 0.35% mean relative abundances (MRA) in the total bacterial community from CR and RP at the flowering stage, respectively. Five known methanotrophic genera were found in rhizospheric soils (Fig. 5). Different methanotrophic genera dominated rhizospheric methanotrophic communities. Those from the CR rotation system were dominated by the alphaproteobacteria *Methylocystis* (84.16% MRA), followed by the gammaproteobacteria *Methylobacter* (10.07% MRA). Conversely, the alphaproteobacterial genus *Methylocella* (51.82% MRA) dominated CR methanotrophic communities, followed by *Methylocystis*-affiliated ASVs (36.65% MRA). The *Crenothrix* genus, belonging to the *Gammaproteobacteria* class, was exclusively retrieved from one replicate of the CR system (3.49% MRA). Although the confirmed methanotroph “*Ca*. Methylomirabilis oxyfera” was not found, the phylum “*Ca*. Methylomirabilota” (formerly phylum NC10) was detected (MRA 0.19%–0.23%) in the total bacterial communities RP and CR rhizospheric soils (data not shown). Additionally, methanotrophic members of the family *Methylacidiphilaceae* (*Verrucomicrobia* class) were not detected, nor were ASVs affiliated with Upland Soil Clusters (alpha and gammaproteobacterial USC).

## Discussion

This work investigated the impact of rice crop intensification on the aerobic methanotrophic communities inhabiting rice rhizospheric soils from two contrasting rice cropping systems: continuous rice and a rice-pastures rotation. Although aerobic methanotrophic bacteria play a crucial role in maintaining and enhancing environmentally sustainable rice production systems, information is scarce regarding the impact of these agricultural practices on this relevant microbial guild. The dynamics of *pmo*A abundance and MOP were evaluated in bulk and rhizospheric soils throughout a rice crop cycle. Moreover, the total and active methanotrophic communities in the rhizospheric soils of CR and RP at the flowering stage were assessed using ^13^C-CH_4_ DNA-SIP experiments and 16S rRNA gene amplicon sequencing. Methanotrophic abundances in bulk and rhizospheric soils from the two cropping systems were addressed in soils from pre-seeding to the flowering stage. Bulk and rhizospheric soils had similar *pmo*A abundances throughout the crop cycle, ranging from 5.61 × 10^6^ to 5.88 × 10^7^ copies g^−1^ dw regardless of crop stage. In contrast, differences between *pmo*A abundances in both compartments (Ma et al. 2013) and higher methanotrophic densities in N-fertilized rice rhizospheric soils and no effect of the crop stage were previously reported (Liu et al. 2019). Our findings revealed significantly higher methanotrophic abundances in the CR cropping system compared to RP, regardless of crop stage or soil compartment (bulk or rhizospheric). Limited information is available about the impact of soil use intensification on methanotrophs associated with rice roots or those found in paddy soils. Breidenbach et al. (2017) reported higher *pmo*A abundances in continuous rice roots when compared with a rice-corn rotation system. In contrast, Jiang et al. (2022) found similar *pmo*A levels in paddy soils from RP and rice-wheat cropping systems.

The MOP also showed significant differences throughout the rice crop cycle. Rhizospheric soils had greater MOPs than bulk soils, similar to those reported by other authors (Bodelier et al. 2000, Eller et al. 2005, Ma et al. 2010). A significant statistical interaction was found between crop stage and crop rotation for MOP in bulk and rhizospheric soils. The tillering stage had the highest MOP rates, not only in rhizospheric soils but also in bulk soils. Conversely, other works, including a previous study on this field experiment, reported maximum methane oxidation rates at the flowering stage in bulk (Fernández-Scavino et al. 2022) and rhizospheric soils (Ma et al. 2013), mainly explained by a major release of root exudates at this phenological stage (Aulakh et al. 2001, Eller and Frenzel 2001, Breidenbach and Conrad 2014). Additionally, since a high methane concentration, similar to conditions used by other authors (He et al. 2012a, Szafranek-Nakonieczna et al. 2019, Fernández-Scavino et al. 2022), was used for MOP determinations, the contribution of high-affinity methanotrophs could be underestimated. Other environmental or physicochemical factors may play a key role in modulating methane oxidation at the tillering stage (Sultana et al. 2019). In this regard, a strong positive correlation was observed between N-NH_4_^+^ content and MOP, which could contribute to explaining this finding. Although diverse and contradictory results have been reported regarding the effect of N-fertilization on methane oxidation, several authors reported stimulant effects (Bodelier et al. 2000, Ferrando and Tarlera 2009, Hu and Lu 2015, Liu et al. 2016). In contrast, the MOP showed a negative and moderate, yet significant, correlation with pH (pH values ranged between 4.41 and 5.20). According to Zhao et al. (2020), pH may be a key driving force in selecting for canonical gamma and alphaproteobacterial methanotrophs in rice paddy soils. Other agricultural practices, such as liming, which increases the pH of soils, may drive low-affinity methanotrophic communities in Amazonian pasture soils (Fonseca de Souza et al. 2025). Further studies should be conducted to evaluate the impact of this factor in this agroecosystem. Noticeably, according to the MOP rates retrieved for CR and RP rotations, a significant impact of intensification on the potential activity of methanotrophs was revealed. Other crop rotations, including those with crops other than rice, have also been shown to affect MOP in soils (Kollah et al. 2020).

On the other hand, a microcosm ^13^C-CH_4_ DNA-SIP experiment from slurry incubations of CR and RP rhizospheric soils at the flowering stage was performed. A sharp enrichment of methanotrophs in the heavy fractions (^13^C-H) of ^13^C-CH_4_ slurry incubations was confirmed. To our knowledge, this is the first report to successfully employ DNA-SIP with an ultracentrifugation step using a fixed-angle rotor (30°), thereby broadening the horizons of this technique.

Total active bacterial communities detected by SIP in rhizospheric soils from CR and RP cropping systems were dominated by methanotrophs (>70%) affiliated with the classes *Alphaproteobacteria* (type II) and *Gammaproteobacteria* (type I). Other non-methanotrophic bacteria, such as the methylotroph *Hyphomicrobium*, repeatedly reported by different authors in SIP experiments (Qiu et al. 2008, Dumont et al. 2011, do Carmo Linhares et al. 2021), and bacterial classes with no clear link to methylotrophic processes were also active. The classes *Bdellovibrionia* (linked to Gram-negative predation, Murase and Frenzel 2007) and *Gemmatimonadetes*, were detected in heavy ^13^CH_4_ fractions, as was previously reported (He et al. 2012b, Qiu et al. 2008, Deng et al. 2019). The presence of the family *Gemmatimonadaceae* was positively correlated with the presence of methanotrophic genera in rice soils (Kaupper et al. 2022). Although cross-feeding could be an explanation, the presence of not-yet-described methanotrophic bacteria cannot be ruled out (Deng et al. 2019).

Regarding the active methanotrophic communities, although an enrichment of type I methanotrophs was detected in SIP microcosm incubations, the dominance of type II methanotrophs, led by *Methylocystis*-affiliated ASVs, was enhanced in all fractions, reaching MRA of 90 and 88% for CR and RP, respectively. Other authors have previously reported the predominance of this genus belonging to the *Beijerinckiaceae* family in total (Eller and Frenzel 2001, Shrestha et al. 2008, Shiau et al. 2018) and active (Murase and Frenzel 2007, Sultana et al. 2019, Zhao et al. 2020) methanotrophic communities from paddy soils and also associated with rice roots (Qiu et al. 2009).

Interestingly, among the total *Methylocystis*-affiliated ASVs (27 ASVs), a different distribution pattern was observed. Eight of them were significantly enriched or exclusively found in CR rhizospheric soils, whereas four ASVs were detected in those from RP. These intra-genus differences could imply an impact of rice intensification, favoring a differential association with rice roots of distinct *Methylocystis* spp. worth exploring. The genus M*ethylocystis* comprises several facultative methanotrophs that can utilize not only one carbon compound as an energy source and carbon source, but also other carbon-carbon bond compounds, mainly acetate (Farhan Ul Haque et al. 2020). On the other hand, *Methylosinus*, also belonging to the family *Beijerinckiaceae*, was also a dominant active methanotroph in CR and RP rhizospheric soils (3.7%–4.9%), which has been found in paddy fields by several authors (Ma et al. 2013, Lüke et al. 2014, Shiau et al. 2018, Jiang et al. 2022). To our knowledge, the detection of this genus as part of the active methanotrophic community in SIP experiments has not been previously reported.

As previously discussed for MOP results, physicochemical factors such as pH can drive type II dominance or the proportion of different types of methanotrophs. Accordingly, in our study, the *Methylocystis* enrichment could have been favored in RP soil incubations under more acidic conditions. Conversely, methanotrophs of type I were found to be the main ones responsible for *in situ* methane oxidation in the rice rhizosphere (Qiu et al. 2008) or were active, as indicated by the PLFA-SIP experiment from the rice rhizosphere (Shrestha et al. 2008). In the present work, type I methanotrophs were active and enriched in heavy fractions of the SIP experiment, although with mean relative abundances <3%. *Methylobacter* (gammaproteobacterial methanotroph) was active in both cropping systems with similar mean relative abundances (1.63% and 1.78%).

Different crop rotations and/or intensification have been shown to impact the structure of soil bacterial communities (Xuan et al. 2012) or those associated with crop roots (Breidenbach et al. 2017), as well as soil methanotrophic communities (Sengupta and Dick 2017, Jiang et al. 2022).

Despite methane fluxes were not measured in the present work, previous studies on the same field experiment reported that CH_4_ was emitted from rice soils only under flooded conditions reaching maximum rates at the flowering stage (Fernández-Scavino et al. 2022). Interestingly, even though no significant differences in methane fluxes were found in rice soils from continuous rice, rice-pasture, and rice-soybean cropping systems, methane cycle populations from bulk soils showed to respond to rice intensification, particularly at the tillering stage (Fernández-Scavino et al. 2022). However, information on their effect on active root-associated bacterial communities or microbial guilds of environmental relevance, such as methanotrophs, is lacking.

Noticeably, differences were detected in the active rhizospheric methanotrophic communities from CR and RP cropping systems. Some methanotrophs were differentially enriched in CR and RP rhizospheric soils, as determined by the differential abundances analysis performed on 13-H-CH_4_ fractions. *Crenothrix, Methyloparacoccus*, and Ca. *Methylospira* were differentially enriched in active methanotrophic communities from CR rhizospheric soils, while *Methyloterricola* and *Methyloglobulus* significantly increased in those from RP. These taxa corresponded to type I methanotrophs, which, although they were not the dominant methanotrophs in the ^13^C-H fractions, have been found to actively contribute to the methane oxidation potential of soils, regardless of their abundance (Shiau et al. 2018).

Active aerobic methanotrophic populations detected by DNA-SIP were compared to the total methanotrophic communities inhabiting rhizospheric soils from CR and RP at flowering.

Methanotrophic sequences represented 1.1% and 0.35% of the total rhizospheric bacterial communities from CR and RP, respectively, similar to what has been reported for rice soils by other authors (Eller and Frenzel 2001, Macalady et al. 2002, Lee et al. 2015). Both rice cropping systems shared four methanotrophic genera: *Methylocystis, Methylocella, Methylobacter*, and *Methylobacterium*; whereas *Crenothrix* was exclusively detected in the CR rhizospheric soils. Surprisingly, according to 16S rRNA gene amplicon sequencing, different profiles of dominant methanotrophs were associated with non-incubated rhizospheric soils from the low (RP) and high (CR) intensive rice cropping systems at the flowering stage. *Methylocella*-affiliated ASVs dominate RP rhizospheric soils (MRA 52%), while *Methylocystis*-affiliated ASVs dominate those from CR cropping systems (MRA 84%). The latter genus was also present in RP rhizospheric soils but with a lower MRA (37%), whereas the MRA of *Methylocella* in CR was lower than 7%. Moreover, *Methylocella*, which does not possess particulate methane monooxygenase (pMMO) but soluble methane monooxygenase (sMMO) (Theisen et al. 2005), is also a facultative methanotroph that can utilize acetate, pyruvate, succinate, malate, and ethanol as carbon and energy sources (Farhan Ul Haque et al. 2020) and is widely distributed (Rahman et al. 2011). Additionally, this genus exhibits low growth rates (Farhan Ul Haque et al. 2020), and it has been proposed that this fact may explain the underestimation of its role in active communities in SIP experiments (He et al. 2012a, McDonald et al. 2005). Thus, versatile facultative methanotrophs would dominate the rhizospheric aerobic methanotrophic communities from these contrasting rice rotation systems. Moreover, these two dominant genera can fix atmospheric nitrogen (Dedysh et al. 2004). Furthermore, nitrogenase structural genes have recently been reported in most type I and II methanotrophic genomes (Hara et al. 2022). Being facultative methanotrophs and providing plant-assimilable nitrogen are outstanding traits that could explain their successful association with rice roots.

Further studies, including those conducted across different rice seasons, should be undertaken to confirm the long-term effects of these two contrasting rice production systems on methanotrophic communities and the impact of environmental factors.

## Conclusions

This work focused on a scarcely addressed, environmentally relevant topic linked to the impact of land use intensification on methanotrophic communities associated with rice roots. A sustainable, low-intensive rice-pastures rotation, with more than 30 years of use, was contrasted with a high-intensive continuous rice cropping system, in a long-term field experiment. A holistic approach was employed, combining various methodologies (*pmo*A qPCR, MOP rates, ^13^CH_4_ DNA-SIP, and 16S rRNA gene amplicon-sequencing). Our findings revealed that methanotrophic communities from bulk and rice rhizospheric soils are dynamic throughout the crop season and are greatly impacted by rice crop intensification. The highest methanotrophic potential (in terms of abundance and potential activity) was observed at the tillering stage, a few days after flooding. Although type II methanotrophs, potentially facultative and diazotrophs, dominated rice rhizospheric methanotrophic communities at the flowering stage in both rice cropping systems, different total methanotrophic assemblages were associated with rice roots at this stage. While *Methylocella* dominated the rice-pastures rhizospheric soils, *Methylocystis* prevailed in those from the CR cropping system. These findings suggest that rice plants grown in low- and high-intensity production systems recruit distinct rhizospheric methanotrophic communities during flowering, when maximum methane fluxes have been previously reported, which may influence methane emissions in these agroecosystems. Furthermore, the ^13^CH_4_ DNA-SIP experiment unveiled that, although *Methylocystis* (a type II methanotroph) dominates active methanotrophic communities from both systems, distinct type I methanotrophs were differentially enriched in active communities from rice pastures (*Methyloterricola* and *Methyloglobulus*) and CR (*Crenothrix, Methyloparacoccus*, and Ca. *Methylospira*). Future research should also consider the roles of other microbial guilds involved in the methane cycle, including anaerobic methanotrophs and methanogens, as well as the influence of environmental factors and management practices (e.g. leaving rice straw on the soil surface under no-till conditions). Such insights are essential for a comprehensive understanding of microbial dynamics and methane emissions as rice cultivation intensifies.

## Supplementary Material

fiaf112_Supplemental_File

## Data Availability

The 16S rRNA gene amplicon sequencing data underlying this article are available in NCBI Sequence Read Archive (SRA) at https://www.ncbi.nlm.nih.gov/sra/, and can be accessed with the BioProject number PRJNA1210543.
