# Supplementary material for: Intensive rice cropping drives shifts in abundance, activity, and assembly of root-associated methanotrophic community
Source: FEMS Microbiol Ecol. 2025 Nov 13;101(12):fiaf112. doi: 10.1093/femsec/fiaf112 (PMC12663087; doi:10.1093/femsec/fiaf112)

**FEMS Microbiology Ecology Journal**

Supplementary Material

**‘Intensive rice cropping drives shifts in abundance, activity, and assembly of root-associated methanotrophic community’**


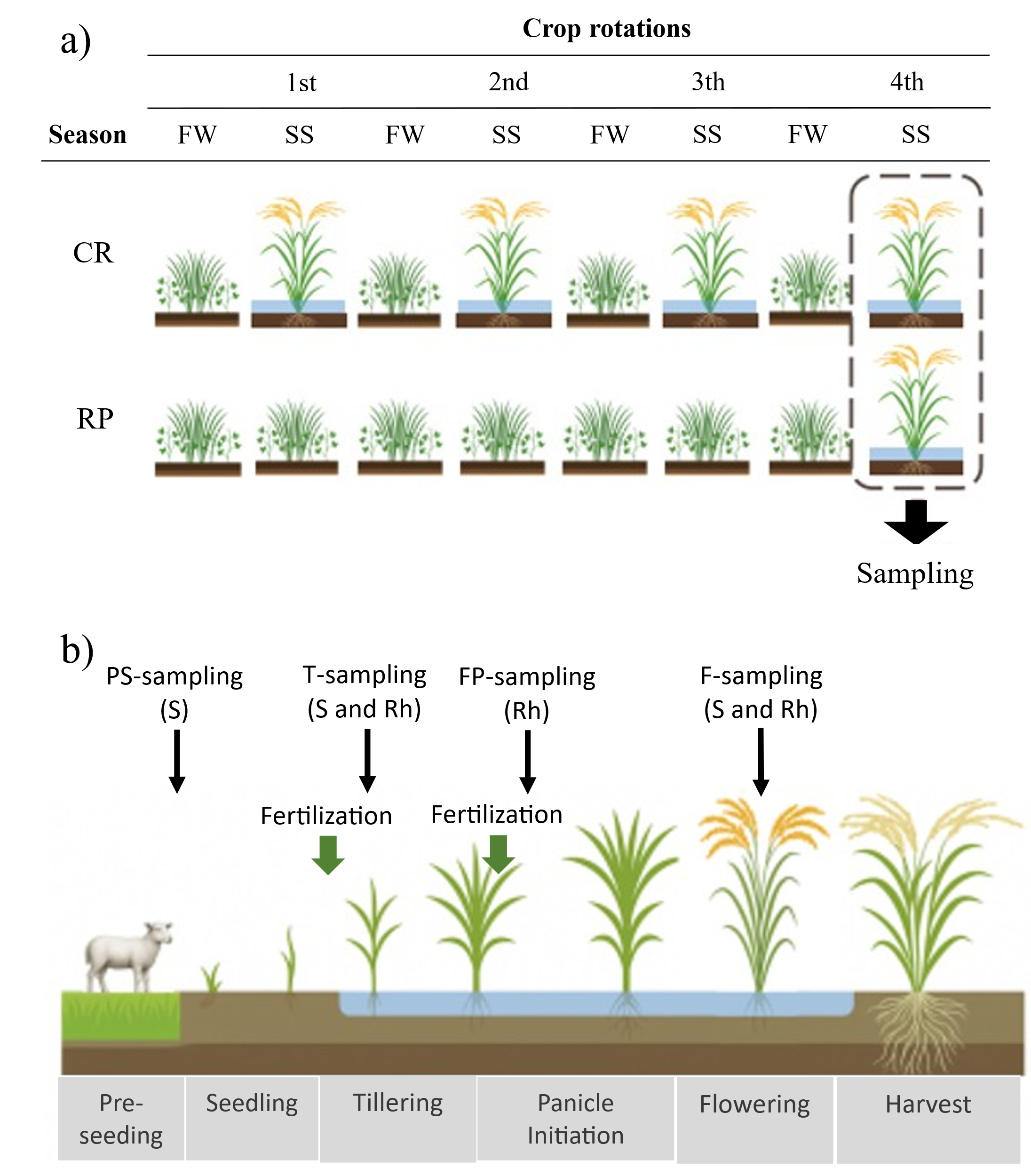


**Fig. S1. Rice field experiment, cropping cycle, and sampling strategy.** Rice-based rotation systems and the rotation phases (a). CR: rice during spring-summer every year; RP: the first rice after the perennial pastures (mix of pastures, refer to M&M section) from a rice-pastures rotation. SS=Spring-summer FW=Fall-winter. An outline of rice cropping and sampling points are also shown (b). Green arrows correspond to nitrogen fertilization events. Samplings throughout the crop cycle of Bulk soil (S) and or Rhizospheric soil (Rh) are indicated. PS: Pre-Seeding; T: Tillering; PI: Panicle Initiation; F: Flowering

**Table S1** Physicochemical properties of bulk and rhizospheric soils from Continuous Rice (CR) and Rice-Pastures (RP) rotation systems at the different stages (PS: pre-seeding; T: Tillering; PI: Panicle Initiation; F: flowering). Results are presented as the mean ± standard deviation of biological replicates (n = 3). nd: not determined.

| Compartment | Crop Rotation | Crop stage | N-NH_4_^+^ (mg/kg) | POX-C ^a^ (mg/kg) | Humidity (%) | pH |
| --- | --- | --- | --- | --- | --- | --- |
| Soil | CR | PS | 7.6 ± 0 .6 | 501 ± 65 | 21.3 ± 2.3 | 4.87 ± 0.31 |
| Soil | RP | PS | 8.6 ± 0.3 | 514 ± 33 | 23.3 ± 0.4 | 4.58 ± 0.05 |
| Soil | CR | T | 27.6 ± 1.7 | 444 ± 99 | 22.5 ± 2.4 | 4.79 ± 0.28 |
| Soil | RP | T | 20.7 ± 9.3 | 517 ± 36 | 25.5 ± 0.9 | 4.65 ± 0.05 |
| Rhizosphere | CR | T | 31.2 ± 13.0 | 537 ± 107 | 24.6 ± 3.8 | 4.61 ± 0.12 |
| Rhizosphere | RP | T | 15.4 ± 5.5 | 523 ± 42 | 26.6 ± 0.2 | 4.41 ± 0.05 |
| Rhizosphere | CR | PI | 9.2 ± 2.2 | 493 ± 60 | 23.5 ± 3.0 | 4.79 ± 0.29 |
| Rhizosphere | RP | PI | 9.7 ± 0.3 | 477 ± 17 | 26.3 ± 0.4 | 4.77 ± 0.14 |
| Soil | CR | F | 4.9 ± 0.8 | 486 ± 67 | 22.9 ± 3.3 | 5.20 ± 0.27 |
| Soil | RP | F | 5.8 ± 0.2 | 440 ± 9.1 | 25.8 ± 0.5 | 5.19 ± 0.10 |
| Rhizosphere | CR | F | 4.3 ± 0.3 | 418 ± 10 | 19.0 ± 0.7 | 4.62 ± 0.17 |
| Rhizosphere | RP | F | 4.4 ± 0.5 | 477 ± 37 | 23.9 ± 1.0 | 4.42 ± 0.14 |

^a^ Permanganate Oxidable Carbon


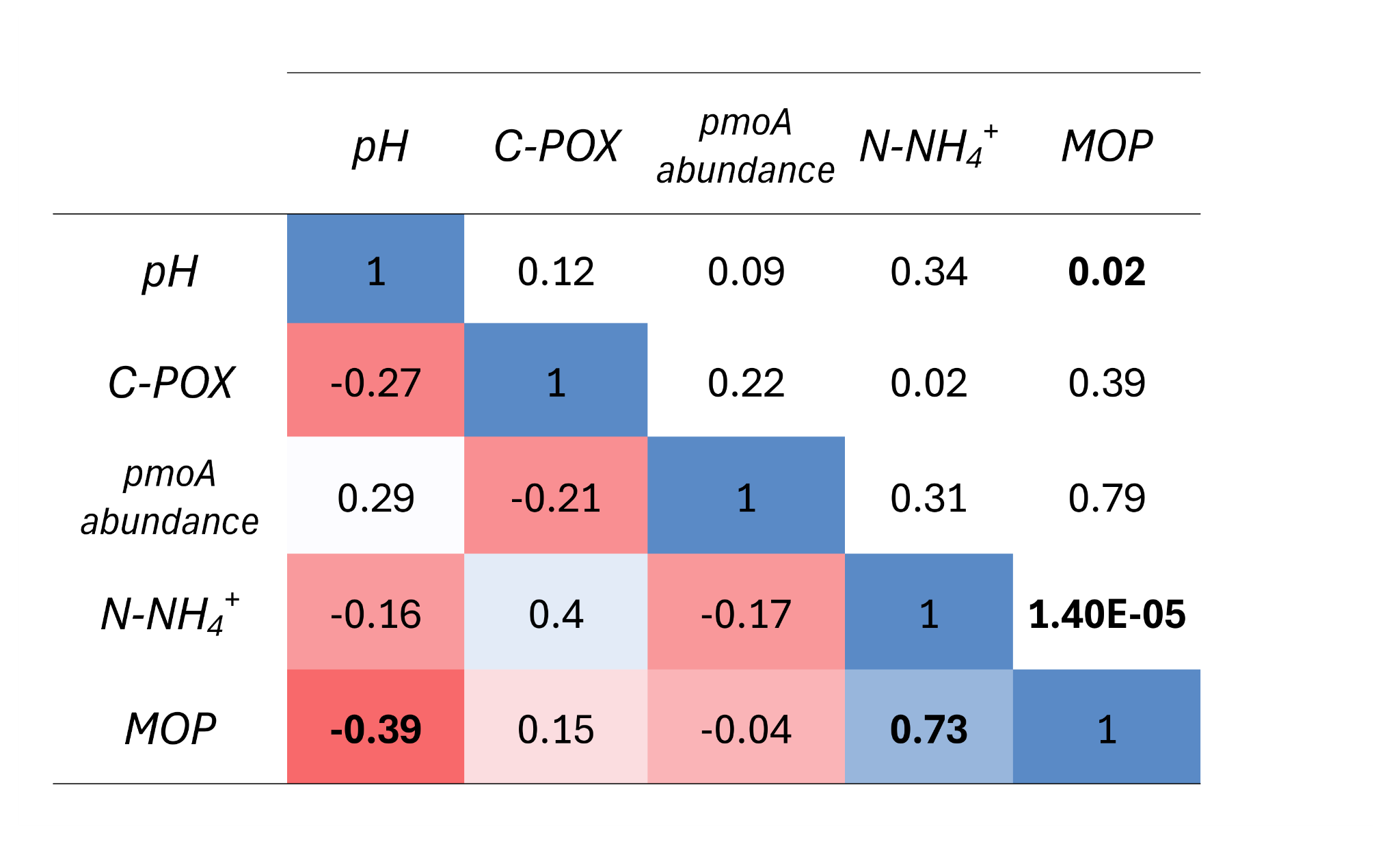


**Fig S2** Spearman’s correlation coefficients among soil physicochemical properties Methane Oxidation Potential (MOP) and *pmo*A abundance. C-POX: Permanganate Oxidable Carbon. Correlation coefficients are indicated in colors: the bluer, the stronger the positive correlation, and the redder, the stronger the negative correlation. The p-values are above the matrix diagonal.

**Table S2**. Number of reads (Illumina MiSeq paired-end, 16S rRNA gene, V4 region) after the different processing steps for rhizospheric soils and SIP fractions. CR: continuous rice; RP: rice-pasture; Rh: original rhizospheric soil; 12: ^12^CH_4_ incubation; 13: ^13^CH_4_ incubation; H: heavy SIP fraction; L: Light SIP fraction; 1 or 2: replicates.

| Sample | Raw reads (paired-end) | Filtered reads | Denoised and merged reads | Non-chimeric reads |
| --- | --- | --- | --- | --- |
| 12_H_CR1 | 92460 | 85334 | 62309 | 59766 |
| 12_H_CR2 | 94999 | 87261 | 61003 | 57543 |
| 12_H_RP.1 | 97639 | 91408 | 66274 | 62145 |
| 12_H_RP.2 | 96168 | 88111 | 60969 | 57453 |
| 12_L_CR.1 | 111606 | 102499 | 71143 | 65082 |
| 12_L_CR.2 | 100905 | 92930 | 62140 | 56789 |
| 12_L_RP.1 | 66338 | 60172 | 40123 | 37635 |
| 12_L_RP.2 | 96245 | 87795 | 59856 | 53775 |
| 13_H_CR.1 | 115985 | 107397 | 91317 | 71368 |
| 13_H_CR.2 | 135230 | 124534 | 103788 | 82068 |
| 13_H_RP.1 | 121533 | 112779 | 95097 | 74548 |
| 13_H_RP.2 | 107316 | 98000 | 81192 | 67494 |
| 13_L_CR.1 | 83178 | 76340 | 52673 | 51494 |
| 13_L_CR.2 | 113415 | 104321 | 72659 | 68732 |
| 13_L_RP.1 | 115260 | 106427 | 73256 | 68502 |
| 13_L_RP.2  RhCR.1  RhCR.2  RhRP.1  RhRP.2 | 115104  68009  105614  84030  103907 | 106994  62732  96875  76352  96311 | 72238  39427  65580  50607  64892 | 67255  38327  63249  46793  59390 |


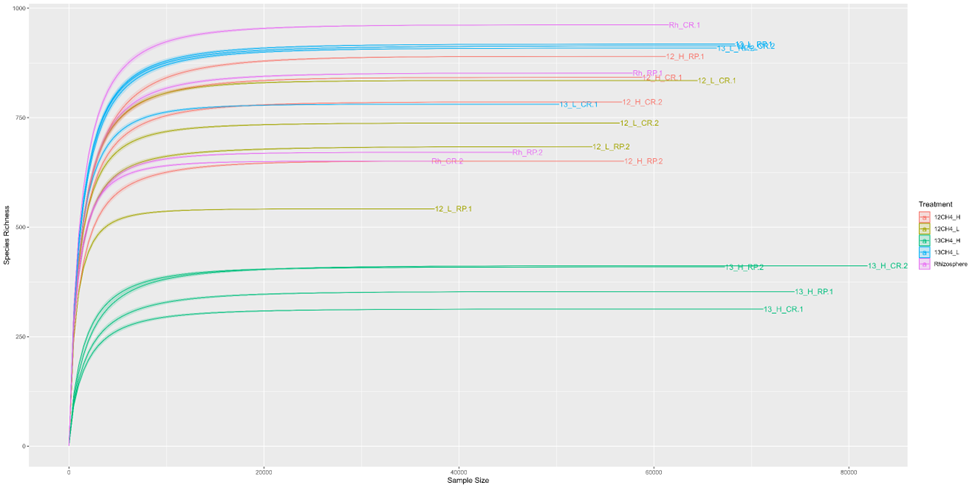


**Fig S3**. Diversity coverage obtained by 16S rRNA gene Illumina MiSeq sequencing for rhizospheric soils (Rh_CR and Rh_RP) and the heavy and light fractions of ^13^CH_4_ SIP- Experiment.


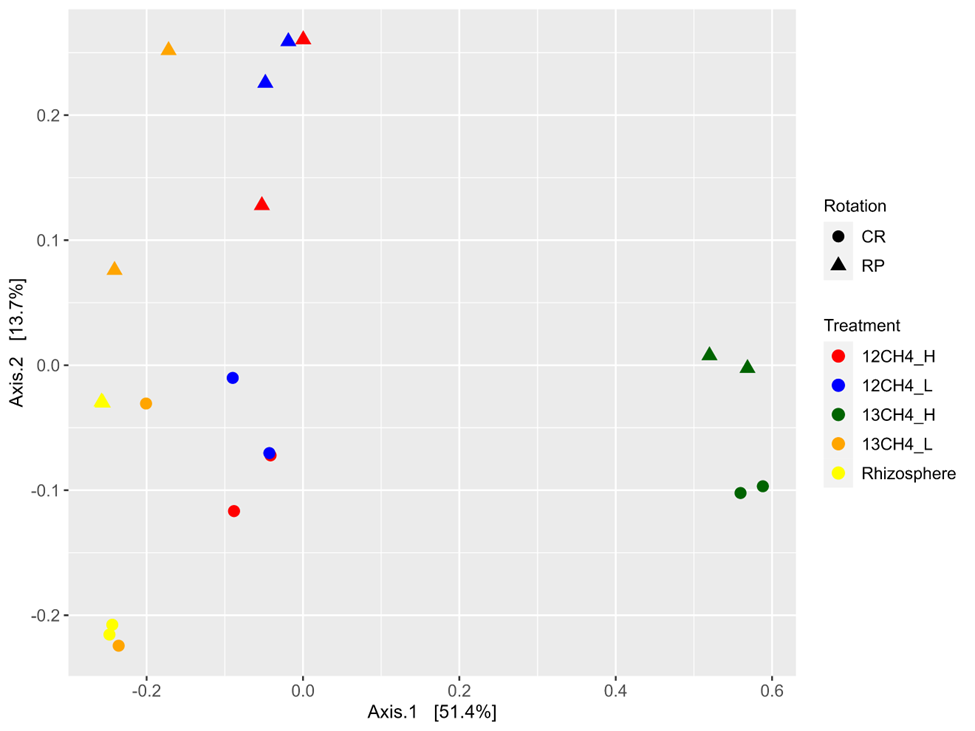


**Fig S4.** Active bacterial communities from DNA-SIP experiment retrieved by 16S rRNA gene amplicon-sequencing of heavy (13_H and 12_H) and light (13_L and 12_L), and from original rhizospheric soils (Rh). PCoA analysis supported by ANOSIM (p<0.01) analysis.

**Table S3**. Diversity indices retrieved from 16S rRNA gene amplicon-sequencing of selected labeled (13) and unlabeled (12) SIP fractions from ^13^C-CH_4_ and ^12^C-CH_4_ (control) slurry incubations, and rhizospheric soils (Rh) for both rotation systems (CR: continuous rice; RP: rice- pastures cropping system). The numbers 1 or 2 correspond to the replicate. Different letters indicate significant differences (ANOVA and Test of Tukey, p-value< 0.01 for each index comparison)


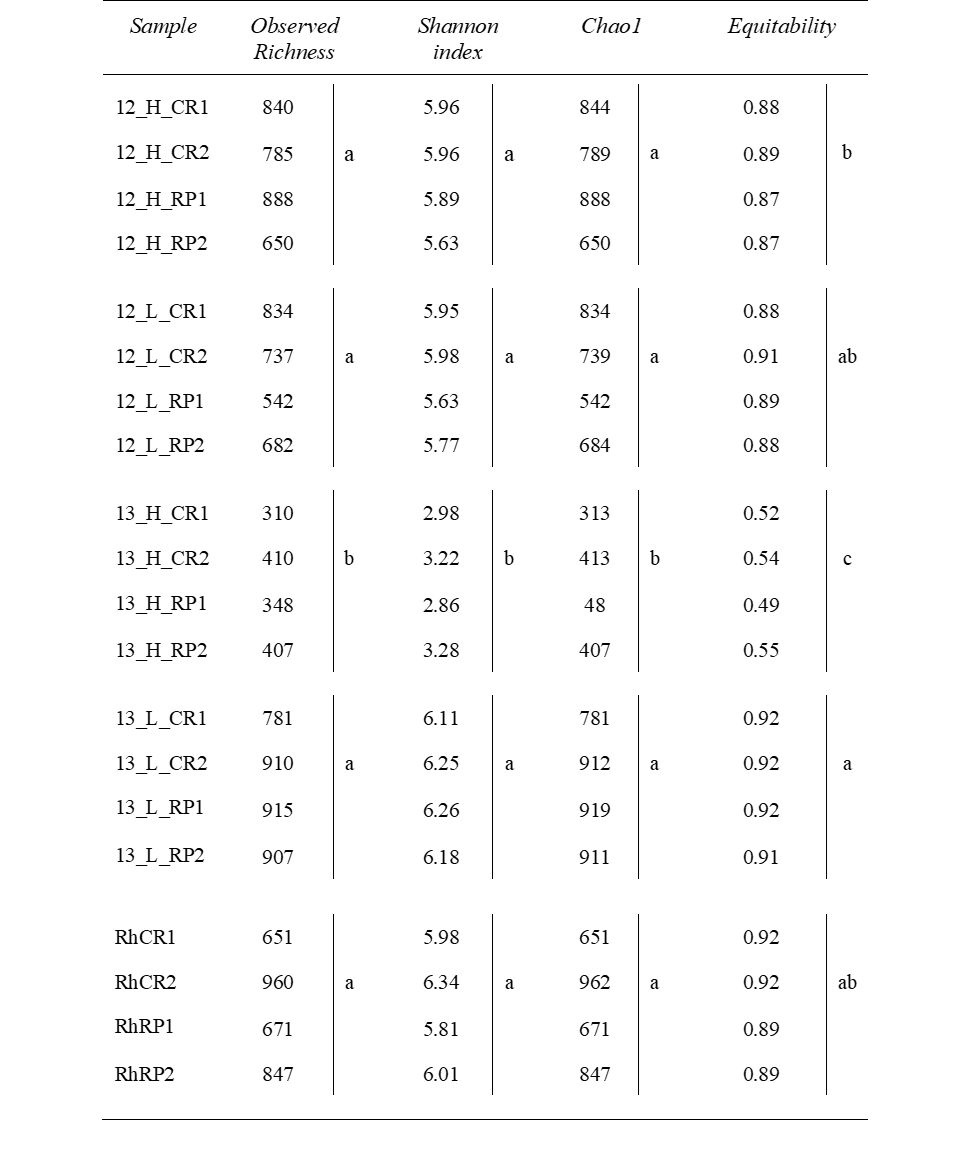

Supplement: fiaf112_Supplemental_File [file fiaf112_supplemental_file.docx]
